# Exploring a complex constellation of signaling pathways

**DOI:** 10.7554/eLife.105095

**Published:** 2024-12-11

**Authors:** Nathaniel C Nelson, Matthias C Kugler

**Affiliations:** 1 Division of Pulmonary, Critical Care and Sleep Medicine, Department of Medicine, New York University Grossman School of Medicine New York United States

**Keywords:** myofibroblast, bronchopulmonary dysplasia, hyperoxia, lung development, postnatal, Mouse

## Abstract

Cells called alveolar myofibroblasts, which have a central role in the development of the lung after birth, receive an orchestrated input from a range of different signaling pathways.

**Related research article** Khan IS, Molina C, Ren X, Auyeung VC, Cohen M, Tsukui T, Atakilit A, Sheppard D. 2024. Impaired myofibroblast proliferation is a central feature of pathologic post-natal alveolar simplification. *eLife* **13**:RP94425. doi: 10.7554/eLife.94425.

Our capacity to perform heavy physical exercise is influenced by many factors, including the total surface area of the alveoli in our lungs. A new-born baby typically has about 50 million lung alveoli, giving a surface area of 10 m^2^ (which is the size of a small kitchen), and these numbers increase to about 300 million and 70–90 m^2^ (the size of a badminton court) in adults. Astonishingly, this expansion occurs ‘silently’ during the first 5–10 years of our lives while we are already breathing. However, any injury to the lungs during this critical period of development will compromise lung function later in life.

This is illustrated by bronchopulmonary dysplasia, the most common lung disease in premature infants. In this disease postnatal lung injury impairs formation of the alveoli and vessels, distorting lung structure and resulting in significant perinatal morbidity and mortality (Jobe and Bancalari, 2001). Moreover, patients with bronchopulmonary dysplasia who reach adulthood have altered lung structure, decreased lung functional capacity and increased susceptibility to lung disease (Northway et al., 1990; Wong et al., 2008). Experimental models of the disease – including hyperoxia (exposure to higher-than-normal levels of oxygen), mechanical ventilation and genetic knockouts in mice – have revealed molecular and cellular mechanisms underlying normal lung development and injury, but much remains unknown (Ahlfeld and Conway, 2012).

The fourth and last stage of lung development requires the subdivision of the simple alveolar sacs present at birth into a complex structure containing ducts and multiple mature alveoli (Figure 1). This involves three different cell types – epithelial cells, mesenchymal cells, and endothelial cells – collaborating with each other in the formation of elongated ‘secondary’ structures that protrude from the walls of the alveolar sacs. Mesenchymal cells called alveolar myofibroblasts have a central role in this process: positioned at specific sites in the alveolar sacs, these cells initiate the formation of the alveolar walls, and remain in place during the elongation process.

**Figure 1.**
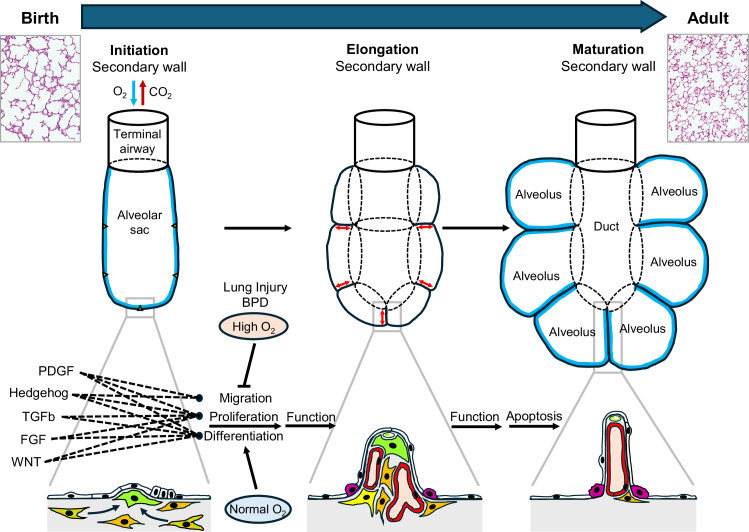
Generation of alveolar surface area in the postnatal lung. At birth, alveolar sacs contain distinct mesenchymal cells (orange and green stripes; left panel), which differentiate into alveolar myofibroblasts (green) to initiate the growth of secondary walls from the primary walls of the sacs at certain points (triangles). This is followed by elongation of these walls (red arrows; middle panel) to form alveoli with entry rings (dotted lines). Each secondary wall contains myofibroblasts at its tip and is covered with alveolar epithelial cells (white and fuchsia). At first the secondary walls also contain abundant fibroblasts (orange) and blood capillaries (red), but they become thinner during maturation and eventually contain only blood capillaries (along with structural molecules such as elastin and collagen); most of the myofibroblasts and fibroblasts disappear by apoptosis. Overall, this process results in a significant increase in the surface area of the alveoli (blue lines; right panel). The change in lung structure can be seen by comparing histology images of a neonatal lung (top left) and an adult lung (top right). During lung development five different signaling pathways (PDGF, hedgehog, TGFb, FGF and WNT; see main text) orchestrate the migration, differentiation and proliferation of alveolar myofibroblasts. However, lung injury around birth (such as bronchopulmonary dysplasia or high levels of oxygen) disrupts this complex constellation of signaling pathways, which leads to compromised lung function later in life. FGF: fibroblast growth factor; WNT: wingless/Int.

Alveolar myofibroblasts also contain contractile elements and produce structural (matrix) proteins that are responsible for ensuring that the adult lung has the elasticity it needs to function properly. To achieve these tasks, myofibroblasts have to migrate, differentiate and proliferate, and these processes are all controlled by signals from surrounding cells. So far researchers have identified five signaling pathways – the PDGF (Li et al., 2018), hedgehog (Yie et al., 2023), TGFb (Sureshbabu et al., 2015), FGF (Li et al., 2017), and Wnt (Li et al., 2020) pathways – that are required for normal myofibroblast function during the formation of alveoli after birth. Moreover, these pathways do not function properly in the lungs of patients with bronchopulmonary dysplasia or in experimental models of the disease. Collectively, these observations highlight the importance of alveolar myofibroblasts.

Now, in eLife, Dean Sheppard and colleagues at the University of California San Francisco – including Imran Khan as first author – report the results of experiments on mice that explore the influence of the TGFb signaling pathway (where TGFb is short for transforming growth factor beta) on alveolar myofibroblasts in both normal and injured lung (Khan et al., 2024). The researchers combined single-cell genetic analyses with two established experimental strategies used to study bronchopulmonary dysplasia.

The first strategy involved inducing lung injury through hyperoxia for several days after birth: this mimics the clinical situation of infants with the disease, who often require mechanical ventilation with high oxygen levels. The second involved knocking out genes that are abnormally expressed in lungs with bronchopulmonary dysplasia, in this case genes associated with the TGFb pathway. This approach tested if the TGFb pathway was required for normal postnatal lung development, and whether its loss protected or worsened lung development during hyperoxic lung injury. Single-cell analysis detected changes in gene expression in multiple cell types, including alveolar myofibroblasts and their progenitors.

Kahn et al. demonstrate how TGFb signaling adds to the complex constellation of signaling pathways that regulates myofibroblasts during the formation of the alveolar wall. While TGFb signaling was required for normal lung development, its loss during hyperoxia was detrimental to alveolar formation and the proliferation of alveolar myofibroblasts, suggesting that it also serves a protective role during lung injury. Single-cell sequencing revealed that although alveolar myofibroblasts in injured lungs show increased TGFb signaling and decreased cell proliferation, disruption of this signaling pathway did not rescue alveolar formation: this suggests that TGFb is part of a failed response to injury rather than being causative.

To gain deeper insight into the cells responsible for TGFb-mediated effects, Kahn et al. used NicheNet, an algorithm that predicts the likelihood of signaling events between different cell types in single-cell gene expression datasets (Browaeys et al., 2020). They showed that altered TGFb signaling and hyperoxia decreased expression of several other signaling pathways vital to these cells, including the PDGF (short for platelet-derived growth factor) and hedgehog pathways. The researchers confirmed this crosstalk by showing that the loss of TGFb signaling in cells that receive PDGF or hedgehog signals worsened alveolar formation and decreased myofibroblast proliferation. This is intriguing since both PDGF and hedgehog signaling are required for the proliferation and differentiation of alveolar myofibroblasts, and the timely interaction between these two pathways is thought to have a role in the initiation and elongation of the alveolar wall (Yie et al., 2023). Conversely, inhibition of PDGF signaling in hedgehog recipient cells has been shown to decrease expression of elastin assembly and TGFb signaling. Collectively, these findings highlight the complexity of signals that converge on myofibroblasts to orchestrate the timing of their appearance and also their function in increasing the surface area of the alveoli in the lung.

It is tempting to envision that it might be possible to boost the proliferation and differentiation of alveolar myofibroblasts – and hence the alveolar surface area – in patients with postnatal lung injury by stimulating one or more of these signaling pathways. However, several hurdles remain. For example, the absence of unique expression markers hampers the identification of alveolar myofibroblasts and their progenitors (Riccetti et al., 2020). Moreover, as shown above, given the complexity of signaling events between different cell types in the developing three-dimensional lung tissue, it will take time to delineate the order, magnitude and combination of signaling factors required to generate a controlled response that is supportive of alveolar growth.
